# Exercise training improves metabolic and circulatory function in COPD patients with NAFLD: evidence from clinical and molecular profiling

**DOI:** 10.3389/fmed.2025.1660072

**Published:** 2025-08-29

**Authors:** Yi-cai Qian, Xiaoxiang Liu, Panpan Liu, ShuYing Jia, Yi Ding, Cuiling Zhu, Jing He

**Affiliations:** ^1^Department of Neurosurgery, Anhui Zhongke Gengjiu Hospital, Hefei, China; ^2^Department of Radiology, First People's Hospital of Changde, Changde, China; ^3^Department of Pulmonary and Critical Care Medicine, Shanghai Pudong New Area Gongli Hospital, Shanghai, China; ^4^Department of Pulmonary and Critical Care Medicine, The Second Hospital of Jilin University, Changchun, China; ^5^Department of Endocrinology and Metabolism, Shanghai Tenth People’s Hospital, Tongji University School of Medicine, Shanghai, China; ^6^Department of Endocrinology, The Third People’s Hospital of Hefei, Hefei Third Clinical College of Anhui Medical University, Hefei, China

**Keywords:** chronic obstructive lung disease, non-alcoholic fatty liver disease, exercise training, transcriptomics, EGR1

## Abstract

**Background:**

Non-alcoholic fatty liver disease (NAFLD) and chronic obstructive pulmonary disease (COPD) are frequently comorbid, affecting up to 41% of COPD patients. Both conditions exhibit significant metabolic and inflammatory dysregulation. While exercise training improves individual disease outcomes by reducing systemic inflammation, its therapeutic effects and underlying mechanisms in COPD patients with NAFLD comorbidity are not well understood. This pilot trial aimed to evaluate the impact of exercise on COPD patients with and without NAFLD, identifying potential biomarkers of exercise-induced regulation.

**Methods:**

COPD patients, categorized by NAFLD status, participated in a 12-week exercise training program. Rehabilitation efficacy was assessed based on lung function and cardiopulmonary exercise test. Metabolic improvements were tested by measuring inflammatory factors, anti-inflammatory factors, etc., using ELISA and PCR methods. Transcriptomic analysis was performed on patients’ samples before and after exercise, integrating external datasets to identify key molecular changes affecting both liver function and systemic inflammation. Hub genes were selected through bioinformatics analysis and validated for their expression in patient samples.

**Results:**

Exercise training elicited beneficial adaptations in both groups. Notably, the COPD with NAFLD group exhibited a greater improvement trend in circulatory function and respiratory metabolic rate compared to COPD-only patients. However, the difference did not reach statistical significance (*p* > 0.05). Furthermore, post-exercise analysis indicated a more pronounced anti-inflammatory shift in the COPD+NAFLD group, with broader reductions in pro-inflammatory cytokines and upregulation of IL-4 compared to the COPD-only group. Transcriptomic analysis integrated with public datasets identified Early Growth Response 1 (EGR1) as a key exercise-response hub gene; its expression was downregulated in the COPD+NAFLD group after exercise but upregulated in the COPD-only group, correlating positively with M2 macrophages (derived from liver single-cell transcriptomic data) and negatively with monocytes.

**Conclusion:**

Our preliminary findings suggest that exercise training may offer unique benefits for patients with comorbid COPD and NAFLD, particularly by enhancing metabolic efficiency and circulatory function. EGR1 may serve as a potential biomarker for exercise responsiveness in this population, reflecting underlying immunomodulatory mechanisms. This insight may also aid in distinguishing COPD subtypes and tailoring exercise-based therapeutic strategies.

## 1 Introduction

Non-alcoholic fatty liver disease (NAFLD) is a chronic liver disorder characterized by excessive fat accumulation in the liver (1, 2), currently recognized as one of the most prevalent liver diseases worldwide. It is closely associated with insulin resistance, obesity, inflammation, oxidative stress, and metabolic syndrome, particularly prominent in middle-aged and elderly populations. Similarly, chronic obstructive pulmonary disease (COPD), which ranks as the third leading cause of death globally, shares the same pathogenic mechanisms partially with NAFLD (3, 4). Both of them lack effective treatment options beyond symptom management, leaving a significant unmet need.

Increasing evidence links NAFLD to pulmonary complications. NAFLD is also recognized as a significant comorbidity in COPD, with its presence associated with the severity of the disease (5). Research has shown that approximately 10% of patients with NAFLD have COPD. Conversely, up to 41% of COPD patients are affected by Metabolic dysfunction–associated steatotic liver disease (MASLD) (1–3), highlighting a significant comorbidity burden. This high comorbidity suggests that COPD and NASLD may share common pathophysiological mechanisms, including chronic systemic inflammation, oxidative stress, and metabolic dysregulation. These interconnected pathways contribute to disease progression and highlight the need for interventions targeting both pulmonary and hepatic health. One proposed mechanism is the “spill-over” theory, which posits that oxidative stress and systemic inflammation in specific COPD subtypes may contribute to liver inflammation, leading to NAFLD. Additionally, COPD patients often exhibit increased visceral fat, which releases free fatty acids that can accumulate in the liver, promoting NAFLD. Nocturnal hypoxia, whether directly related to COPD or linked to comorbid obstructive sleep apnea (OSA), may further trigger NAFLD development (6). Despite these connections, the role of NAFLD in COPD remains underexplored, indicating a gap in research that requires more focused studies on the interplay between these conditions.

Exercise training has been widely recognized as a beneficial intervention for both COPD (4–7) and metabolic disorders, offering systemic anti-inflammatory effects, improving insulin sensitivity, enhancing lipid metabolism, and strengthening respiratory function. Given the shared inflammatory and metabolic pathways in COPD and NAFLD, exercise training may be a promising strategy to mitigate disease progression in patients with this comorbidity. Recently, one study (8) has demonstrated that exercise training can alleviate pulmonary abnormalities, including inflammation and fibrosis, in a mouse model of High-Fat, High-Carbohydrate induced NAFLD by enhancing mitochondrial function. This suggests that exercise may play a therapeutic role in managing respiratory complications associated with metabolic liver diseases. Furthermore, numerous studies have indicated that exercise training may be beneficial in managing NAFLD (9–12), as it enhances fat metabolism, reduces hepatic fat accumulation and inflammation, improves insulin sensitivity, promotes weight loss, and mitigates oxidative stress. Therefore, we hypothesize that exercise training can effectively reduce systemic inflammation in patients with comorbid COPD and NAFLD, and its effect may be greater than in individuals with COPD alone.

To address this hypothesis, we conducted this pilot study to evaluate the therapeutic effects of personalized exercise interventions, identify key molecular targets involved in disease modulation, and provide scientific evidence for the development of targeted treatment strategies.

## 2 Materials and methods

### 2.1 Patients and exercise training protocol

This was a pilot exploratory study conducted without *a priori* effect size estimation, and a formal sample size calculation was not performed, as the sample size was determined by the availability of eligible participants during the study period. The primary purpose was to generate preliminary data to inform the design of future adequately powered studies.

Six COPD patients with NAFLD (COPD+NAFLD) and six COPD patients without NAFLD(COPD-only) were selected from the clinical cohort study at Pudong New Area Gongli Hospital (Registration number: ChiCTR2100053232), all of whom were already enrolled in a 12-week individualized exercise training program. The training was conducted on a Cycle Ergometer (Qianjing 20,003, China) for three days a week, with adaptive loads determined by the results of the cardiopulmonary exercise test (CPET) and continuous functional tests. The exercise intensity was individually calculated based on CPET parameters, with a load adjustment of Δ50% load ±10 Watts [Δ50% load = load at anaerobic threshold – increasing load per minute×0.75/2 + (peak load – increasing load per minute×0.75)/2]. The detailed protocol can be found in our previously published article (13). A sample calculation is provided in Supplementary Table S2 to illustrate how the Δ50% workload was derived. Each session included 30 min of effective exercise at a constant pedaling rate of 55 revolutions per minute (RPM), followed by a cool-down period until the patient’s heart rate returned to baseline.

This study was approved by the ethics committee of Shanghai Pudong New Area Gongli Hospital [2020(14)], and all participants provided written informed consent for the use of their blood samples in scientific research, following the Declaration of Helsinki.

### 2.2 Calculation of emphysema CT score

The calculation of the emphysema CT score is performed using ITK-SNAP for image segmentation and quantitative analysis. First, import the patient’s pre- and post-exercise CT scan data (DICOM format) and use ITK-SNAP’s manual or semi-automatic segmentation tools to label the emphysema regions. In manual segmentation mode, use the Brush Tool or Polygon Tool to label each slice. In semi-automatic mode, apply the Active Contour Segmentation method to optimize the selection. After segmentation, use ITK-SNAP’s Statistics tool to extract the area (𝐴𝑖) of the emphysema region in each slice and calculate the total volume. Finally, calculate the CT score using the following formula: Score= $\frac{\sum_{i=1}^{n}S_{i}\times A_{i}}{N}$ , where 𝑆𝑖 represents the score for each slice, 𝐴𝑖 is the area of the corresponding region, and 𝑁 is the total number of slices.

### 2.3 Sample preparation, RNA isolation, and sequencing

Three milliliters of fresh whole blood were collected from patients before and after exercise. Within two hours of collection, peripheral blood leukocytes were isolated from the blood using Pancoll gradient centrifugation from a single Vacutainer EDTA tube. The isolated cells were then frozen in liquid nitrogen and stored at −80°C for subsequent analyses and PCR. Total RNA extraction was performed utilizing the TRIzol reagent, following the manufacturer’s protocol. Four samples from participants were selected to construct libraries for transcriptome sequencing. The libraries were sequenced on the Illumina HiSeq X Ten platform. All sequencing processes were conducted by OE Biotech Co., Ltd. (Shanghai, China).

### 2.4 Construction of WGCNA network

The WGCNA R package was used to construct a weighted gene co-expression network (WGCNA). The top 5,000 genes with the highest variance were selected for further analysis. Pairwise Pearson correlation coefficients between genes were calculated and transformed into a weighted adjacency matrix, with a soft threshold set to 10 to ensure the network exhibited scale-free topology. The weighted adjacency matrix was then converted into a topological overlap matrix (TOM), allowing for a more accurate estimation of network connectivity by considering both direct and indirect gene interactions. Hierarchical clustering was performed on the TOM matrix to generate a dendrogram, and genes were assigned to distinct modules based on the similarity of their expression patterns, with each module represented by a unique color. These modules group together genes with similar expression profiles, potentially reflecting their involvement in shared biological processes or pathways.

### 2.5 Analysis of immune cell infiltration

The CIBERSORT algorithm was utilized to analyze RNA-seq data from different patient subgroups to estimate the relative proportions of 22 immune cell types. The “corrplot” package was employed to explore interactions among immune cells and assess their potential functional relationships. The “vioplot” package was used to visualize the relative abundance of immune cells, evaluating the impact of gene expression on immune infiltration. Furthermore, Spearman correlation analysis was performed to examine the association between gene expression levels and immune cell infiltration. A *p*-value < 0.05 was considered statistically significant.

### 2.6 Real-time PCR and enzyme-linked immunosorbent assay (ELISA)

For cDNA synthesis, 1 μg of total RNA was reverse transcribed using a reverse transcription kit (Promega, Madison, WI, USA) for mRNA. Real-time PCR was performed using a SLAN-96S PCR System (Bio-) with 2 × SYBR Green PCR Master Mix (Solarbio, Beijing, China). *β*-actin was used as an internal control. The relative expression levels of mRNAs were calculated using the 2^−ΔΔCt method. All experiments were performed in quadruplicate to ensure reproducibility. Primer sequences are listed in Supplementary Table S1. Expression of inflammatory factors like interleukin (IL)-1β, IL-6, IL-8, IL-17, and IFN-*α* in serum was tested utilizing ELISA kits (Sen-Xiong Company, Shanghai, China) following the manufacturer’s protocols.

### 2.7 Statics

Statistical and bioinformatic analyses were conducted using GraphPad Prism (La Jolla, CA, USA) and R software (version 4.3.3; The R Foundation for Statistical Computing, https://www.r-project.org/). For quantitative clinical data, the Shapiro–Wilk test was used to assess normality. Due to the small sample size (*n* = 6 per group), non-parametric tests were primarily employed. Data are presented as medians with interquartile ranges (IQR) for continuous variables, and absolute frequencies for categorical variables. Comparisons between two groups were performed using the Mann–Whitney U test for unpaired data and the Wilcoxon signed-rank test for paired data. For outcomes measured at both time points in each group, a two-way mixed (repeated-measures) ANOVA was applied, with “Time” (pre vs. post) as the within-subject factor and “Group” as the between-subject factor, followed by *post hoc* pairwise tests where appropriate. To control for potential confounding effects of BMI and ALT, multiple linear regression models were performed using both peak VO₂ and delta VO₂ (pre-post exercise difference) as dependent variables. Covariates were selected based on baseline group differences. A *p*-value ≤ 0.05 was considered statistically significant, and all tests were two-sided.

For bioinformatic analysis, differential expression of miRNAs was assessed using the DESeq2 package in R. Genes with an absolute log2 fold change ≥ 1 and a Benjamini–Hochberg adjusted *p*-value < 0.05 were considered significantly differentially expressed.

## 3 Results

### 3.1 Baseline characteristics and preliminary group comparisons

Baseline demographic and clinical characteristics of the study participants are presented in Table 1. At study entry, the two groups, COPD+NAFLD and COPD-only, were broadly comparable across most demographic factors, including age and sex, as well as smoking history, medication use, comorbidities, and various blood cell counts. However, a significant difference was observed in Body Mass Index (BMI), with the COPD+NAFLD group exhibiting a higher median BMI (28.67 kg/m^2^) compared to the COPD-only group (18.74 kg/m^2^; *p* = 0.0043). Additionally, Alanine Aminotransferase (ALT) levels were significantly higher in the COPD+NAFLD group (29.00 U/L) compared to the COPD group (22.50 U/L; *p* = 0.031). No other significant differences were noted in baseline lung function parameters, other liver function markers, lipid metabolism, or emphysema score between the groups.

**Table 1:** Baseline characteristics of COPD patients with and without NAFLD.

	COPD+NAFLD (*n*=6)	COPD (*n*=6)	*P* value
Demographic data			
Sex, male, *n* (%)	5 (83.3)	5 (83.3)	*P* = 1.00
Age, years^†^	64.5 (62–67)	62.5 (59–70)	*P* = 0.91
BMI, Kg/m2^†^	28.67 (25.36–29.38)	18.74 (16.22–19.92)	*P* = 0.0043**
Smoking history			
Current smoker (n)	1	0	*P* = 1.00
Ex-smoker (*n*)	5	6	
Smoking, pack-years^†^	12.5 (3–30)	22.5 (15–35)	*P* = 0.410
Lung function, median (IQR)†			
FEV1/pred, %	38.55 (34.40–41.20)	28.80 (24.50–53.70)	*P* = 0.410
FEV1/FVC, %	48.20 (41.00–54.32)	43.85 (37.00–46.19)	*P* = 0.650
RV/TLC, %	69.77 (46.75–75.00)	49.00 (0–74.50)	*P* = 0.190
Dlco /pred, %	50.00 (40.00–64.00)	49.0 (IQR: 0–74.5)	*P* = 0.880
Complication, *n* (%)			
Hypertension	5 (83.3)	4 (66.7)	*P* = 1.00
Osteoporosis	2 (33.3)	5 (83.3)	*P* = 0.242
Lower limb vascular plaque	5 (83.3)	3 (50%)	*P* = 0.545
Inhaled steroids			
Yes	3 (50)	1 (16.7)	*P* = 0.221
Blood cell count (×10^9^/L)^†^			
WBC	6.93 (6.74–7.31)	6.93 (6.36–7.67)	*P* = 0.873
Eosinophil	0.165 (0.145–0.96)	0.10 (0.10–0.14)	*P* = 0.104
RBC	4.18 (3.91–4.27)	4.37 (3.99–4.89)	*P* = 0.730
PLT	236.0 (209.5–256.5)	196.5 (171.0–234.0)	*P* = 0.556
Neutrophil	4.72 (4.52–4.92)	4.14 (3.08–5.21)	*P* = 1.00
Liver function and lipid metabolism^†^			
ALT	20.00 (20.00–20.35)	29.00 (22.50–29.50)	*P* = 0.031**
AST	27.00 (23.25–28.00)	26.50 (24.00–29.50)	*P* = 0.904
Homocysteine	10.80 (10.19–25.97)	10.97 (9.28–12.90)	*P* = 0.660
TG	1.11 (0.88–1.46)	0.72 (0.50–0.78)	*P* = 0.057
TC	4.43 (3.73–4.92)	4.44 (3.71–4.79)	*P* = 0.914
HDL-C	0.94 (0.91–1.10)	1.21 (1.10–1.59)	*P* = 0.400
Emphysema score			
	17.33 (14.11–20.57)	17.16 (10.57–23.75)	*P* = 0.37

^†^Data are presented as median (interquartile range, IQR), **P < 0.05.

To account for baseline imbalance in BMI and ALT, we further assessed their influence on exercise capacity. Multiple linear regression showed that BMI was independently associated with peak VO₂ (*β* = 33.01, *p* = 0.041), consistent with prior evidence (14). However, when delta VO₂ (post-pre) was used to represent training responsiveness, neither BMI nor ALT significantly predicted the outcome (BMI: *p* = 0.8531; ALT: *p* = 0.1624), suggesting these baseline factors did not confound the observed intergroup differences in exercise improvement (Supplementary Tables S3, S4).

^†^Data are presented as median (interquartile range, IQR). Demographic characteristics, smoking history, lung function parameters, complication prevalence, and inhaled steroid use are presented for two groups of COPD patients: those with and without Non-Alcoholic Fatty Liver Disease (NAFLD). *p*-values were computed using Fisher’s exact test for categorical variables and the Mann–Whitney U test for continuous variables. Data are expressed as count (percentage), median (interquartile range, IQR). Abbreviations: FEV1/pred – Forced expiratory volume in 1 s as a percentage of the predicted value; FEV1/FVC – Ratio of forced expiratory volume in 1 s to forced vital capacity; RV/TLC – Ratio of residual volume to total lung capacity; DLco/pred – Diffusing capacity of the lungs for carbon monoxide as a percentage of the predicted value; ALT – Alanine aminotransferase; AST – Aspartate aminotransferase; TG – Triglycerides; TC – Total cholesterol; HDL-C – High-density lipoprotein cholesterol; LDL-C – Low-density lipoprotein cholesterol; WBC – White blood cell count; RBC – Red blood cell count; PLT – Platelet count; Neutrophil – Neutrophil count; Eosinophil – Eosinophil count. RBC (Red Blood Cell count); The CT score of emphysema in the two patient groups shows no significant difference.

### 3.2 Impact of exercise training on lung function, exercise capacity, and cardiorespiratory responses

**Figure 1:**
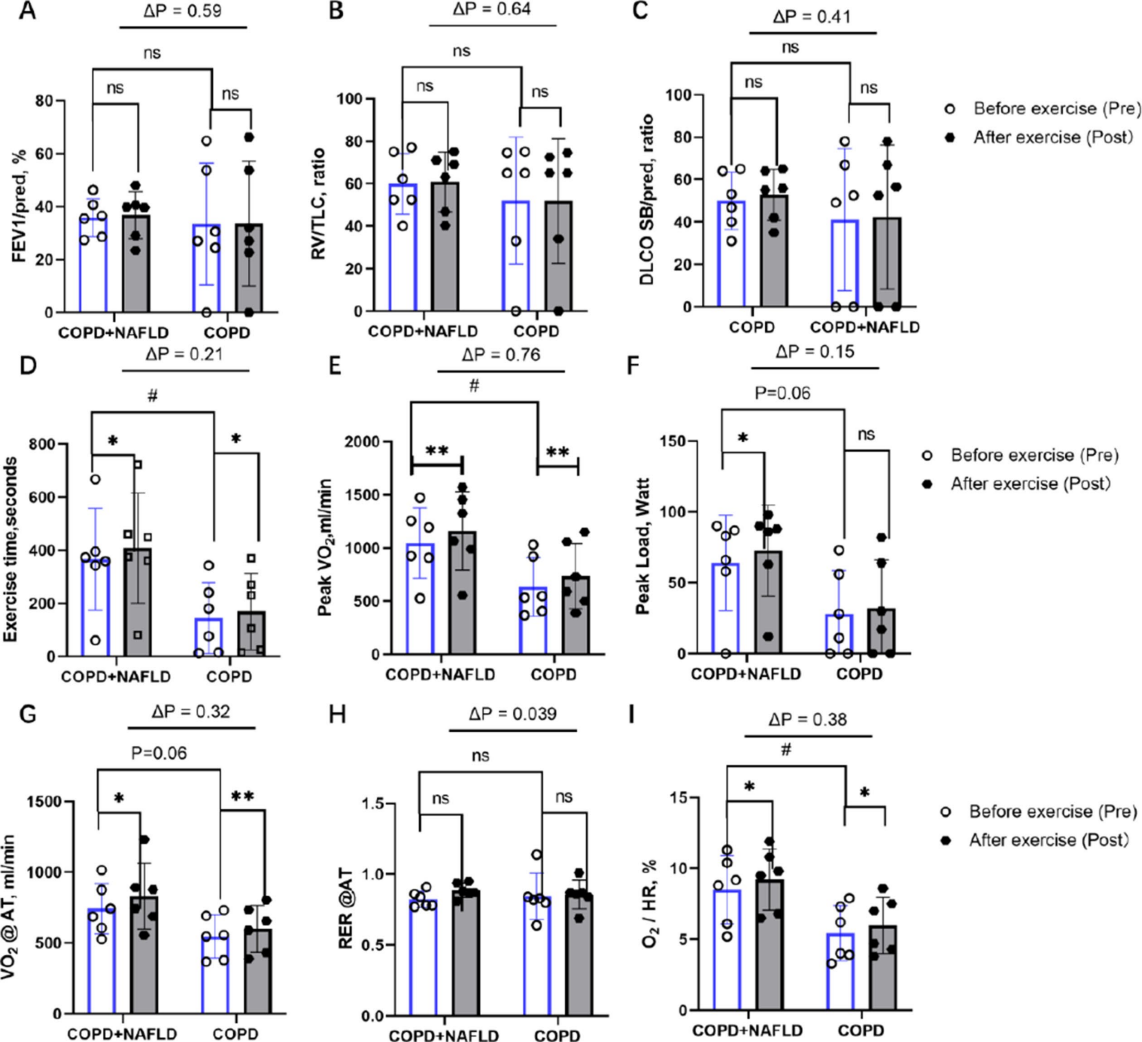
Changes in pulmonary function and cardiopulmonary exercise capacity after exercise. **(A)** FEV₁ % predicted: no significant pre–post change in either group; ΔP not significant. **(B)** Residual volume/TLC ratio: no significant pre–post change in either group; ΔP not significant. **(C)** DLCO SB/pred ratio: no significant pre–post change in either group; ΔP not significant. **(D)** Exercise duration: Significant improvement in both groups; ΔP not significant. **(E)** Peak VO₂: significant improvement in both groups; ΔP not significant. **(F)** Peak workload: Borderline increase in COPD+NAFLD only (*p* = 0.06); ΔP not significant. (G) VO₂ at anaerobic threshold: Significant improvement in both groups; ΔP not significant. **(H)** Resting RER: No significant pre–post change in either group; ΔP significant (*p* = 0.039). **(I)** O₂ pulse: Significant improvement in both groups; ΔP not substantial. *p* values above bars indicate between-group differences in Δ (post–pre) values; *p < 0.05, **p < 0.01 = considerable pre–post change within group; #*p* < 0.05 = considerable baseline difference between groups.

Following 12 weeks of exercise training, neither group showed significant improvements in other pulmonary gas exchange functions, including FEV1%predicted, RV/FVC ratio, or DLCO % predicted (Figures 1A–C). In terms of exercise endurance, both groups demonstrated significant improvements in exercise time (COPD+NAFLD: *p < 0*.05; COPD: *p* < 0.05; Figure 1D) and peak VO₂ (COPD+NAFLD: *p < 0*.01; COPD: *p < 0*.01; Figure 1E). Peak workload showed a borderline increase in the COPD+NAFLD group (*p* = 0.06) but no significant change in the COPD-only group (Figure 1F). The between-group differences in improvement (ΔP) for these exercise capacity measures were not statistically significant. Regarding metabolic efficiency (Figures 1G–H), VO₂ at anaerobic threshold (AT) increased significantly in both groups after exercise (COPD+NAFLD: *p < 0*.05; COPD: *p < 0*.01), with no significant ΔP between groups. The RER at AT showed a significant between-group difference in change (ΔP = 0.039), although no significant pre–post change was observed within either the COPD+NAFLD or COPD-only group. For circulatory function (Figure 1I), O₂ pulse increased significantly in both groups following training (COPD+NAFLD: *p < 0*.05; COPD: *p* < 0.05), with no significant ΔP between groups.

After exercise, both groups showed an increase in cholesterol and triglyceride levels, while low-density lipoprotein (LDL), high-density lipoprotein (HDL), AST, and ALT exhibited a downward trend. However, these changes were not statistically significant. Notably, uric acid levels decreased in the COPD+NAFLD group but increased in the COPD-only group (*p* < 0.05). Exercise training led to substantial improvements in exercise tolerance, oxygen uptake, and circulatory efficiency in both groups, with most outcome measures showing comparable changes between COPD+NAFLD and COPD-only patients. A significant between-group difference was observed only for RER at AT, suggesting potential differences in metabolic adaptation patterns. To further explore these physiological changes, additional analyses were performed on systemic biomarkers to investigate possible underlying mechanisms.

### 3.3 Differential expression genes and enrichment analysis after exercise in COPD+NALFD patients

**Figure 2:**
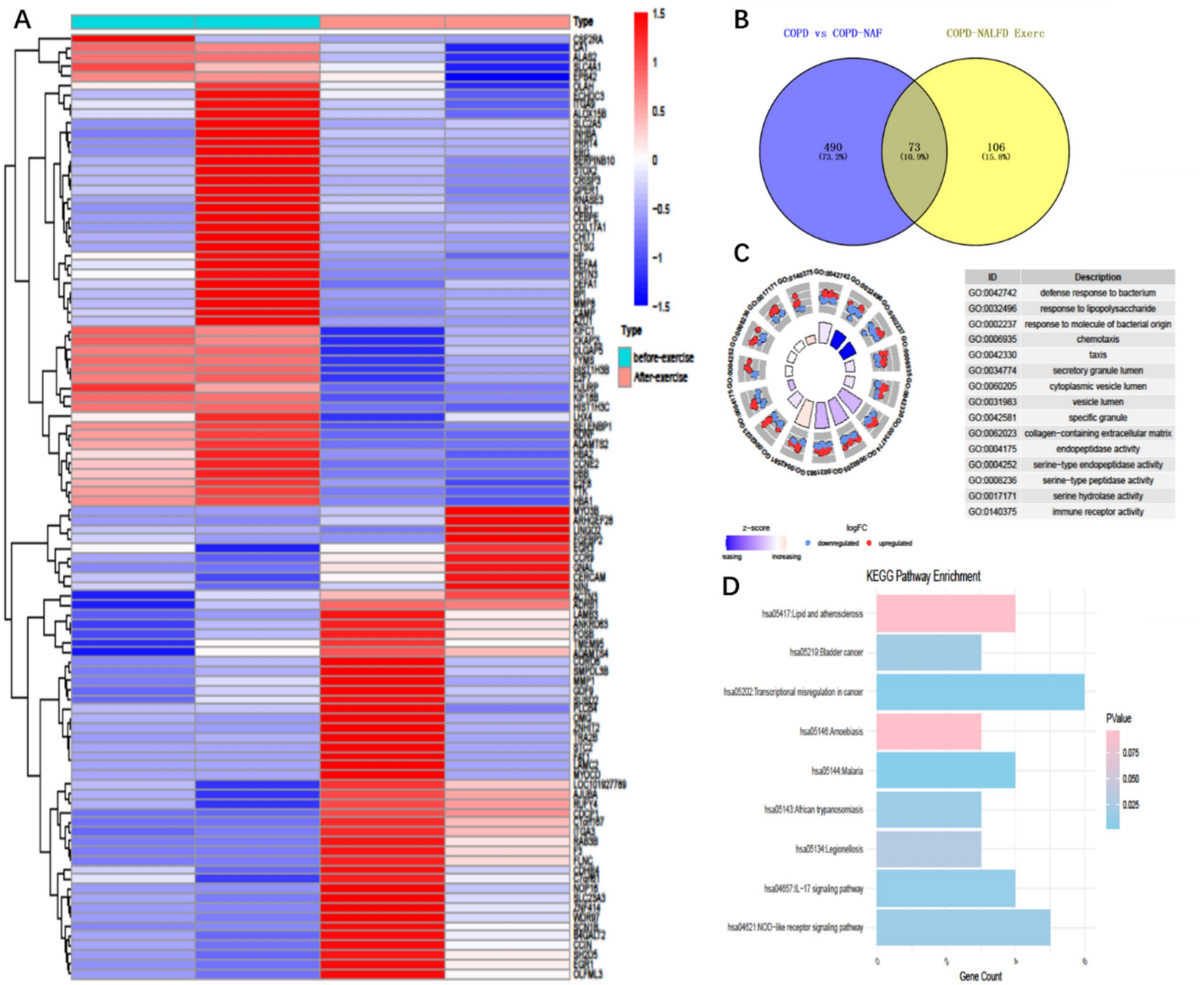
Differentially expressed genes and enriched pathways in COPD patients with NAFLD before and after exercise training. **(A)** 179 Differentially expressed genes (Degs) in COPD+ patients before and after exercise intervention, **(B)** Intersection 179 Degs with 563 Degs between COPD with NAFLD and COPD without NAFLD, resulting in 73 common differentially expressed genes. **(C)** Gene ontology (GO) enrichment analysis of these 73 differentially expressed genes. **(D)** KEGG pathway enrichment analysis of the 73 differentially expressed genes.

Building on our previously published work, we re-analyzed the transcriptomic data of COPD+NAFLD patients. We compared their mRNA profiles before and after exercise training to identify potential biomarkers and underlying mechanisms contributing to the observed differences in exercise responses. Following our screening criteria (log2 FoldChange ≥ 1.0 and *p*-values < 0.05), we identified 178 differentially expressed mRNAs (DE-mRNAs) in the COPD+NAFLD patients, with 70 up-regulated and 108 down-regulated (Figure 2A). Meanwhile, by comparing the transcriptomic data of COPD-only and COPD+NAFLD patients, 562 differentially expressed genes (190 down-regulated and 372 up-regulated) were identified. The intersection of these two datasets revealed 73 differentially expressed genes (Figure 2B). These genes were primarily enriched in pathways related to immune response, inflammation resolution, lysosomal activity, autophagy enhancement, and the mitigation of protease-mediated tissue damage, as well as lipid metabolism, atherosclerosis-related pathways, cancer-related pathways, and the IL-17 and NOD-like receptor pathways (Figures 2C,D). Therefore, we hypothesize that exercise training may improve both liver and overall health by modulating inflammation, enhancing systemic and cellular immunity, and regulating protease activity. To assess changes in inflammation levels, ELISA was performed on peripheral blood samples collected from patients before and after the exercise intervention.

### 3.4 Effects of exercise on serum inflammatory cytokine levels in COPD+NAFLD patients

**Figure 3:**
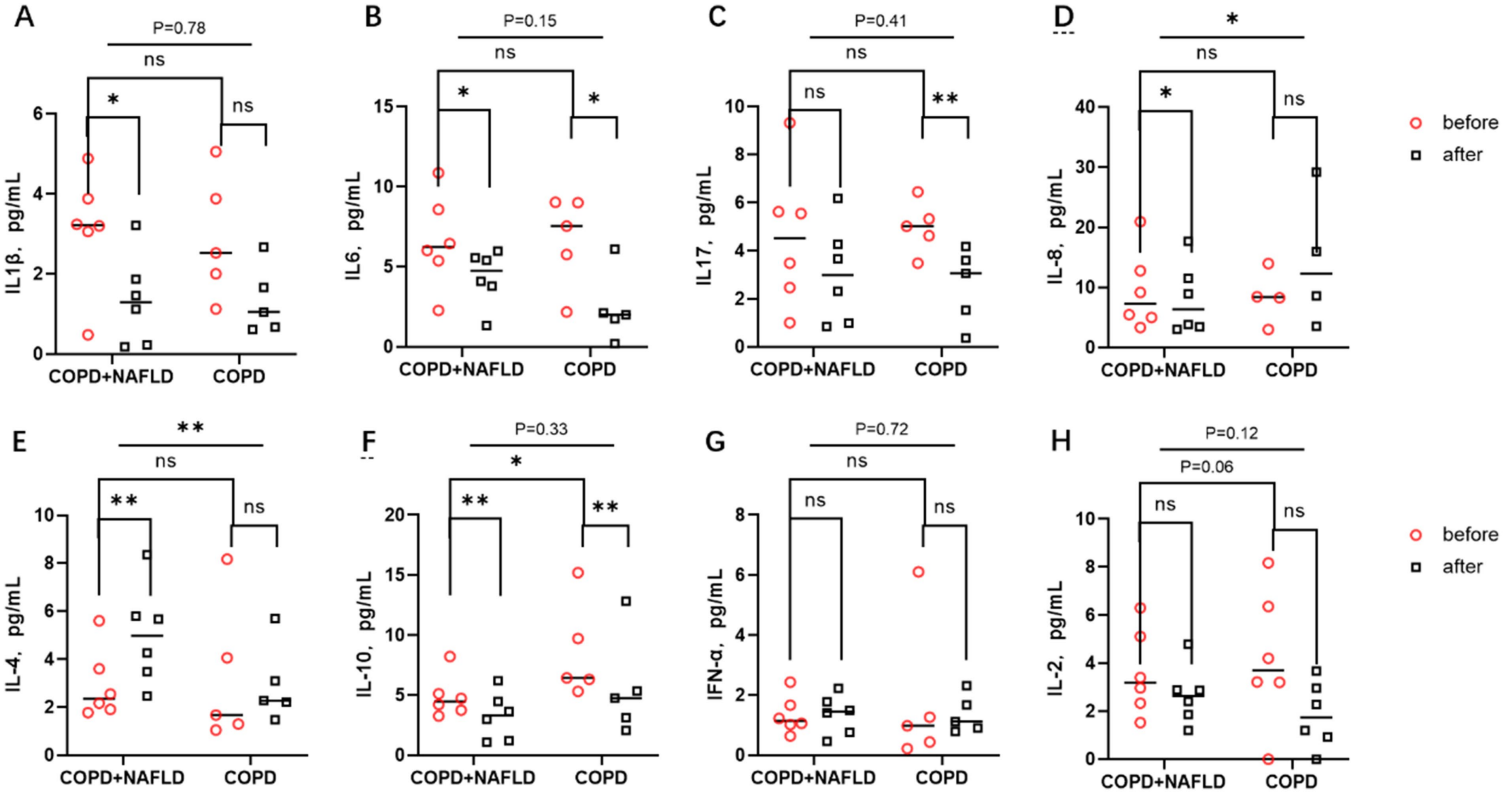
Changes in inflammatory factors before and after exercise in the body. **(A,B)** There were no significant differences in IL-1β and IL-6 levels between the two groups. After exercise, pro-inflammatory factors IL-1β and IL-6 decreased in the COPD-NAFLD group, whereas only IL-6 decreased in the COPD-only group compared to pre-exercise levels. (C) There were no differences in IL-17 levels between the two groups; after exercise, IL-17 levels decreased in the COPD-only group. **(D)** No differences in IL-8 levels were observed between the two groups. Following exercise, IL-8 levels decreased in the NAFLD group, with significant differences in the changes between the two groups. **(E,F)** There were no differences in anti-inflammatory factor IL-4 levels between the two groups, but there was a difference in IL-10 expression; after exercise, IL-4 levels were upregulated in the COPD+NAFLD group. After exercise, IL-10 levels decreased in both groups. **(G,H)** There were no differences in the expression levels of the cell immune factors IFN and IL-2 between the two groups, and their expression trends did not change after exercise. **p < 0.01, *p < 0.05 after exercise vs. before exercise.

Following the exercise intervention, significant reductions in pro-inflammatory cytokines IL-1β and IL-6 were observed in the COPD+NAFLD group, whereas only IL-6 levels decreased in the COPD-only group. IL-17 and IL-8 exhibited different trends between the two groups. After exercise, IL-17 levels only decreased in the COPD-only group, while no significant changes were observed in the COPD+NAFLD group. Conversely, IL-8 levels decreased in the COPD+NAFLD group, but no significant change was detected in the COPD-only group (Figures 3A–D).

For anti-inflammatory cytokines, IL-4 levels increased in both groups after exercise, with a more pronounced increase in the COPD+NAFLD group. In contrast, IL-10 levels decreased after exercise in both groups, with lower expression levels of IL-10 in the COPD+NAFLD group compared to the COPD-only group at baseline. No significant differences were observed in the levels of cellular immune cytokines IFN-*α* and IL-2 between the two groups at baseline, and their expression trends remained unchanged after the exercise intervention(Figures 3E–H). To investigate why exercise training had different effects on systemic inflammatory and anti-inflammatory responses in the two groups, we further analyzed single-cell data from liver tissue transcriptomic datasets to identify specific genes with potential regulatory roles.

### 3.5 Identification of different expression cell types and genes involved in NAFLD from public databases

We identified 27 distinct cell subpopulations within GSE129516 (Supplementary Figures 1A,B), and classified them into eight cell types: endothelial cells, natural killer (NK) cells, macrophages, B cells, monocytes, hepatocytes, T cells, and fibroblasts. In comparing the control and disease groups, the cell types with the most significant differential expression were particularly endothelial cells, macrophages, and monocytes (Supplementary Figures 1C,D). Given the close association between endothelial cells and immune cells in immune response and inflammation regulation, we focused on extracting the marker genes for endothelial cells for further analysis.

**Figure 4:**
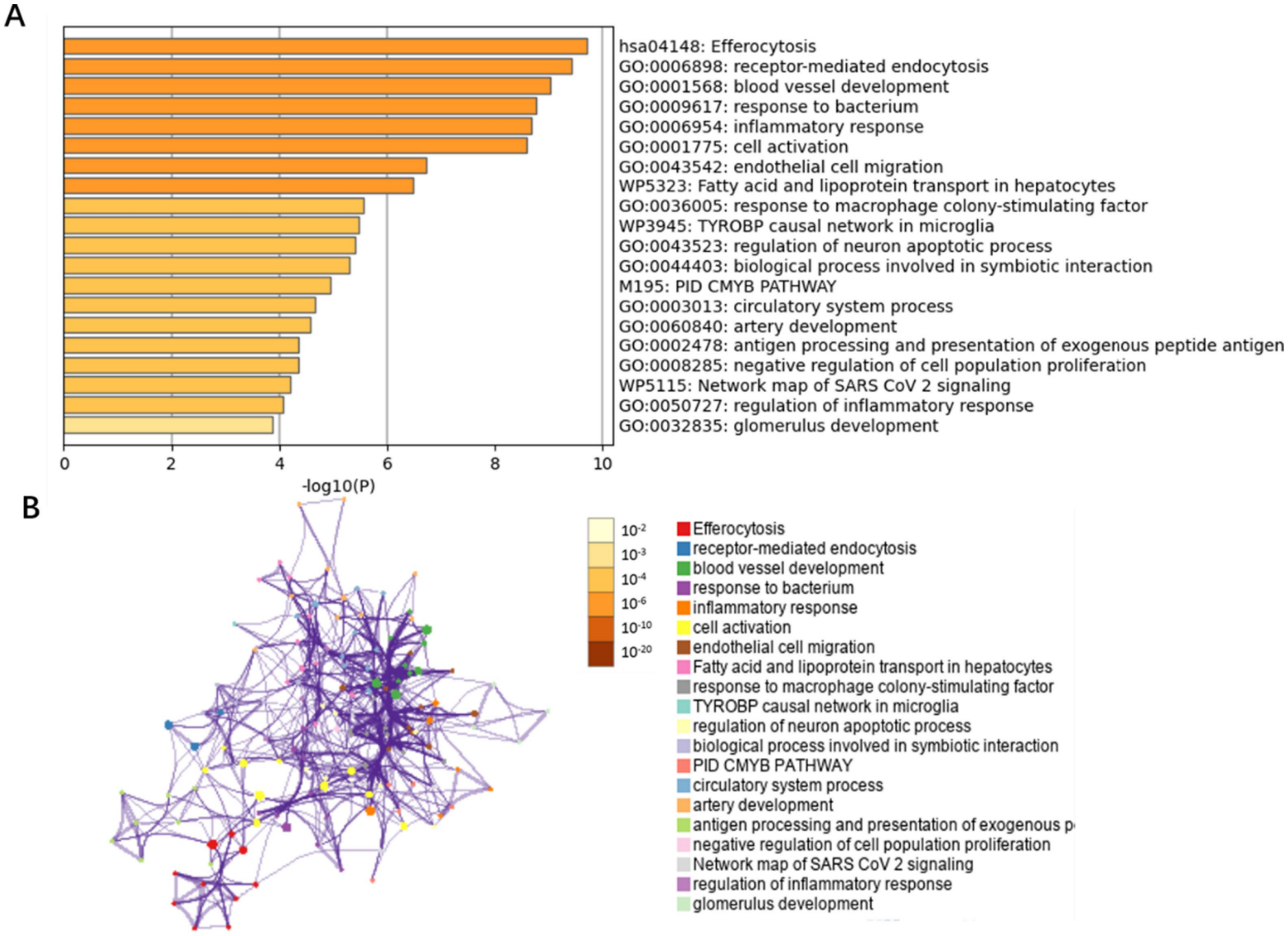
Bar graph of enriched terms across NAFLD-related differentially expressed genes in endothelial cells. **(A)** GO biological functions and pathways enriched for the 49 differentially expressed genes in endothelial cells, colored by *p*-values. **(B)** Network of enriched terms: colored by p-values.

By integrating transcriptomic data from the GSE66676 and GSE126848 datasets, we compared 48 controls and 41 NAFLD patients. 215 differentially expressed genes were identified (adj. *p < 0*.05 and |Log2FC| > 0.585), with 84 upregulated and 131 downregulated. These genes were then mapped to endothelial cell markers, yielding 49 intersecting genes. Metascape database revealed these genes are mainly enriched in inflammatory response, protein catabolic process, angiogenesis, blood vessel development, and immune system regulation pathways (Figure 4).

### 3.6 Construction of a WGCNA network to identify key genes associated with COPD+NAFLD

**Figure 5:**
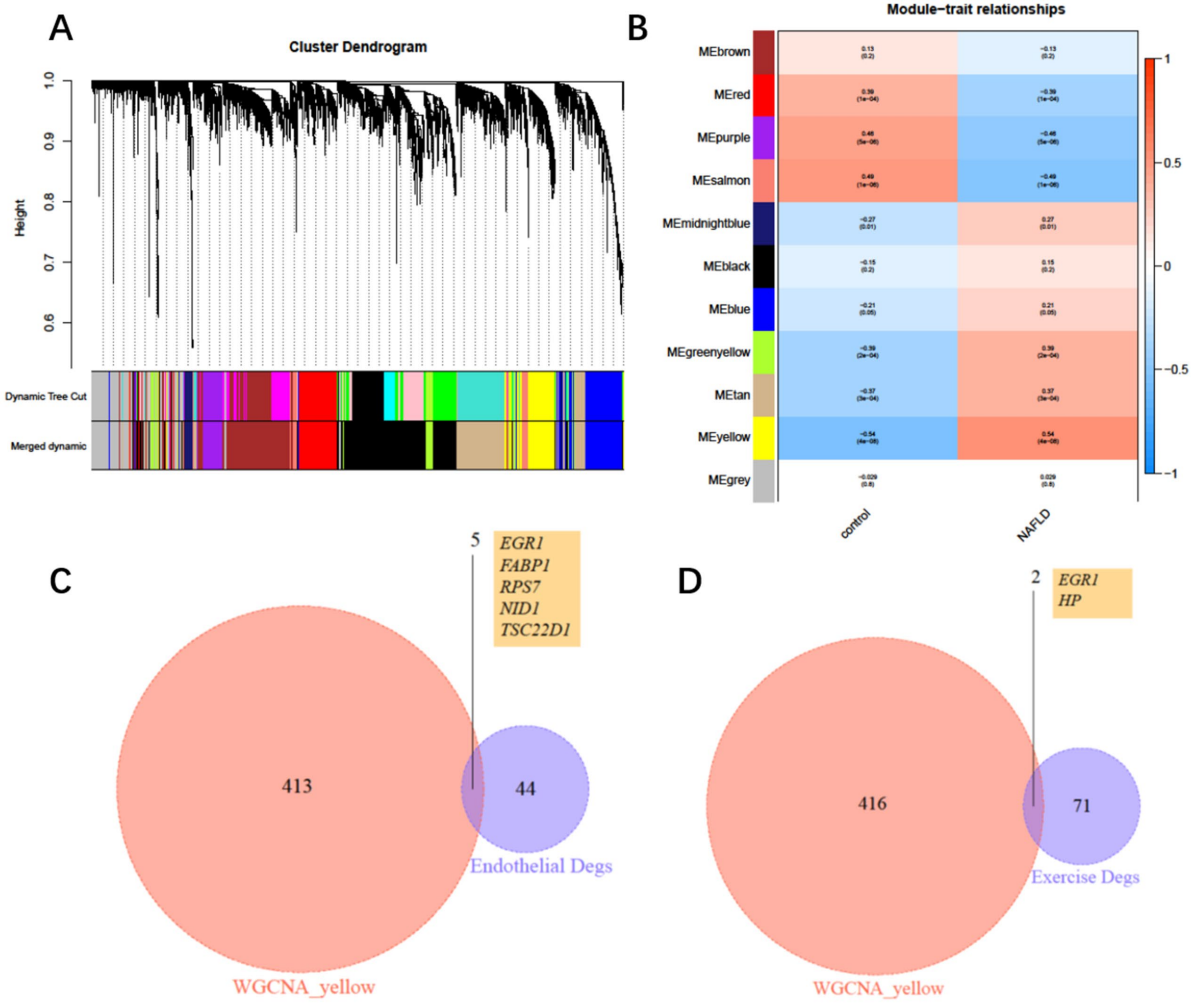
Weighted gene co-expression network and hub genes. **(A)** Clustering dendrogram of consensus module eigengenes. **(B)** Heatmap of the correlation between NAFLD and module eigengenes. **(C)** Intersection of differentially expressed genes (DEGs) from the yellow module and endothelial DEGs. **(D)** Intersection of differentially expressed genes (DEGs) from the yellow module and exercise intervention DEGs.

To further identify key genes through which exercise influences COPD with NAFLD, we constructed a weighted gene co-expression network (WGCNA) based on the merged expression profiles. WGCNA analysis identified a yellow module that was significantly associated with NAFLD, comprising 419 genes (Figures 5A,B). We then intersected these genes with the 50 differentially expressed endothelial cell genes and exercise-mediated differentially expressed genes, ultimately identifying six key genes: EGR1, FABP1, RPS7, NID1, TSC22D1, and HP (Figures 5C,D). Among these genes, EGR1 is expressed in both endothelial cells and peripheral blood leukocytes, and its expression level may be regulated by exercise intervention.

### 3.7 EGR1 as a potential hub gene in exercise-induced immune modulation

**Figure 6:**
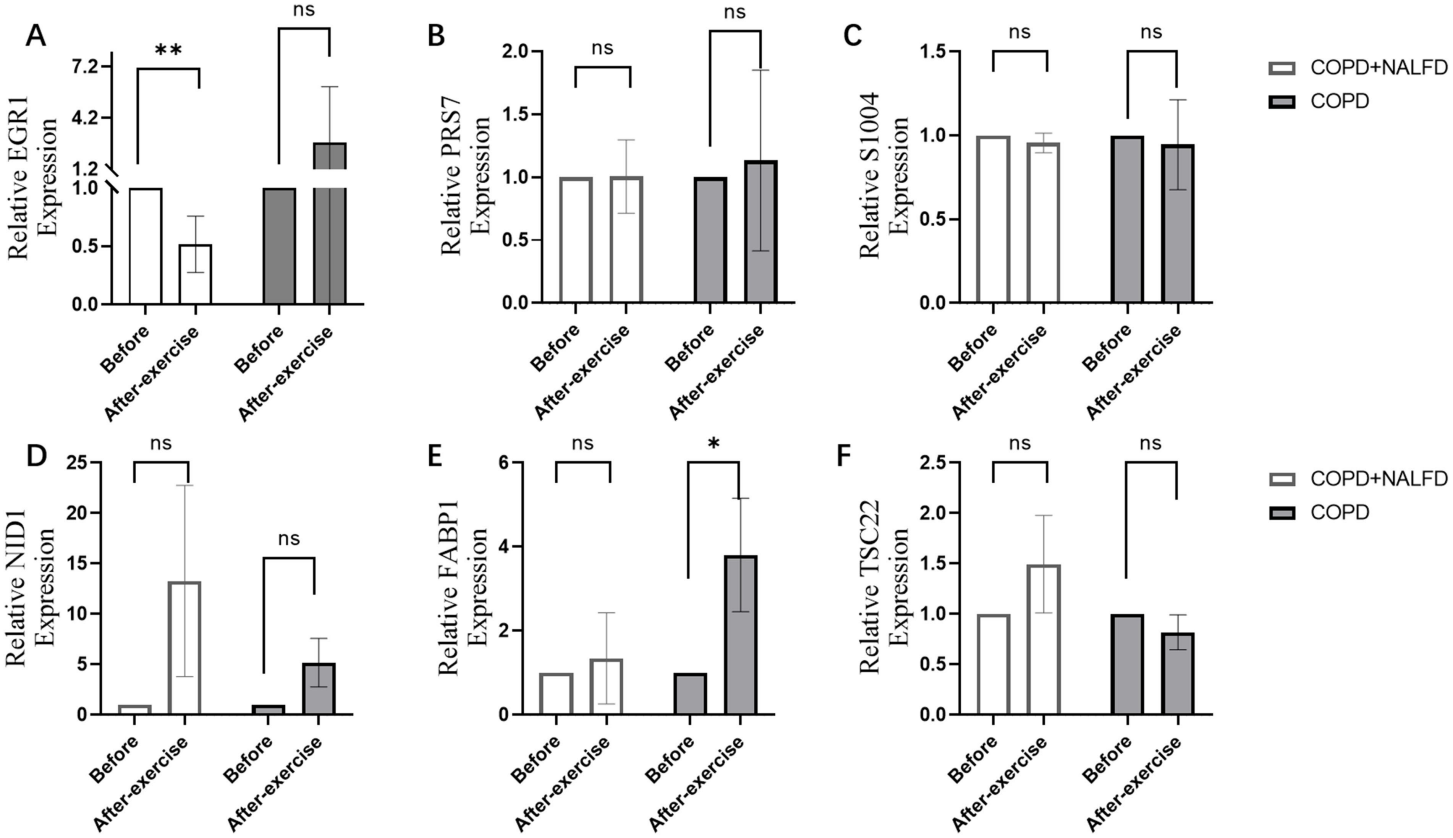
Relative expression level of hub genes after exercise in COPD+NAFLD patients and COPD-only patients. **(A)** In COPD+NAFLD patients, the relative expression level of EGR1 decreased after exercise, with statistically significant differences, whereas it increased with a trend without significance in the COPD-only group. **(B–F)** The relative expression levels of PRS7, S1004, NID1, FABP1, and TSC22 all showed substantial changes after exercise.

After exercise, EGR1 expression decreased in the COPD+NAFLD group, but no changes were observed in the COPD-only group (Figure 6A). In contrast, no significant changes were found in the expression levels of PRS7, S1004, NID1, FABP1, and TSC22 (Figures 6B–F). These results suggest that EGR1 may be a key gene associated with exercise-related changes in the COPD+NAFLD comorbidity.

**Figure 7:**
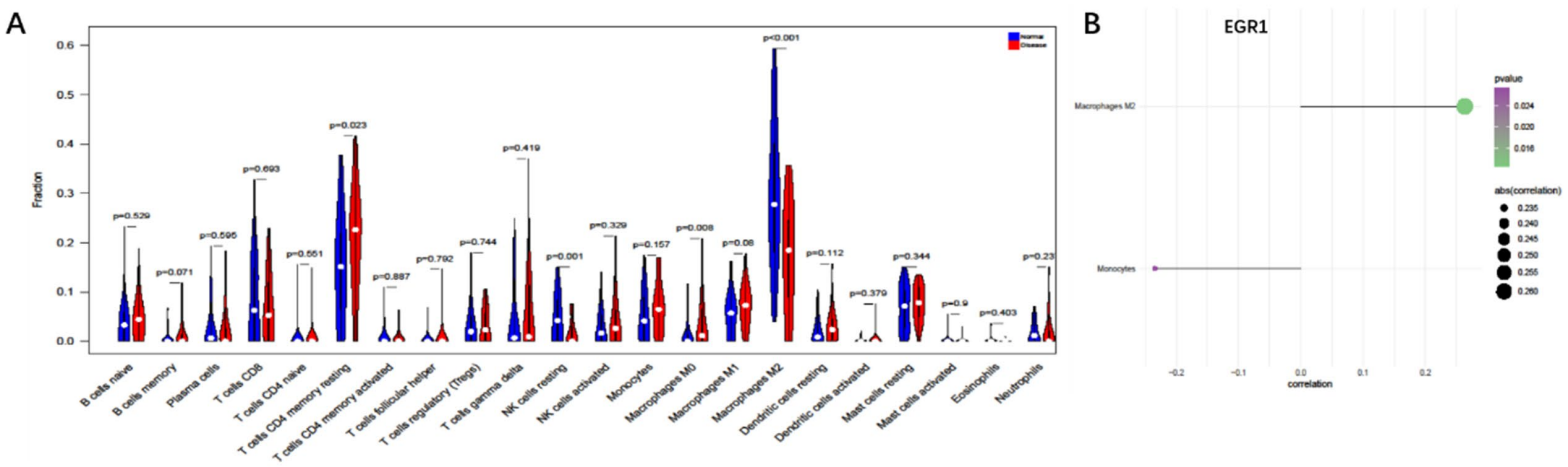
Immune cell infiltration analysis based on liver single-cell data. **(A)** Compared to healthy individuals, NAFLD patients exhibit significantly higher levels of CD4 memory resting T cells and M0 macrophages **(B)**. EGR1 is positively correlated with M2 macrophages and negatively correlated with monocytes.

To further explore the potential mechanisms linking EGR1 to COPD+NAFLD comorbidity, we examined the immune microenvironment in liver tissues. The composition of immune cells in each NAFLD patient is presented in Supplementary Figure 2A, showing significant correlations among different levels of immune infiltration (Supplementary Figure 2B). Compared to healthy individuals, NAFLD patients exhibited significantly higher levels of CD4 memory resting T cells and M0 macrophages, while M2 macrophage levels were significantly reduced (Figure 7A). Furthermore, EGR1 expression was positively correlated with M2 macrophages and negatively correlated with monocytes (Figure 7B). These analyses confirm that EGR1 is closely associated with immune cell infiltration and plays a vital role in the immune microenvironment.

## 4 Discussion

Consistent with existing literature (10), our findings at baseline revealed that patients in the COPD with NAFLD group exhibited significantly higher median ALT levels compared to the COPD-only group (29.00 U/L vs. 22.50 U/L; *p* = 0.031), indicating a pre-existing metabolic disturbance. Although baseline AST levels did not differ significantly (*p* = 0.904), it is well-established that exercise intervention can reduce serum ALT and AST levels and decrease intrahepatic fat accumulation, which aligns with the potential benefits of exercise in our cohort. This is particularly important for COPD+NAFLD patients, who are often prone to more severe metabolic disturbances and inflammation (15). Furthermore, our study demonstrated that following the exercise intervention, COPD+NAFLD patients experienced a significant reduction in circulating pro-inflammatory cytokines (such as IL-1β, IL-6, IL-8, and IL-17) and an increase in anti-inflammatory cytokine IL-4 expression, particularly when compared with COPD-only patients. Although there were no baseline differences in IL-4 levels between the two groups, exercise induced a significant upregulation of IL-4 in the COPD+NAFLD group, suggesting a potentially greater capacity for exercise to enhance Th2-mediated anti-inflammatory responses in this population. In contrast, IL-10 levels decreased after exercise in both groups. While IL-10 is conventionally regarded as an anti-inflammatory cytokine, emerging evidence indicates that it exerts a dual role depending on the pathological context (16): in chronic airway diseases and specific tumor microenvironments, IL-10–mediated immunosuppression may impair pathogen clearance and contribute to persistent inflammation. The reduction in IL-10 observed in our study might therefore reflect a shift toward restoring a more balanced immune response, rather than a simple loss of anti-inflammatory signaling. This interpretation, however, should be viewed with caution given our limited sample size, and warrants confirmation in larger cohorts. These findings suggest that exercise may exert a more pronounced anti-inflammatory and therapeutic effect in individuals with co-existing COPD and NAFLD.

Research indicates that both NAFLD patients and elderly individuals with COPD tend to exhibit higher baseline RER levels compared to healthy controls, often attributed to metabolic disorders such as muscle loss and impaired glycolysis (17–20). These metabolic abnormalities can consequently limit exercise capacity, especially when anaerobic metabolism is critical for energy production. In the current study, COPD+NAFLD patients who completed a 12-week exercise intervention demonstrated a significant increase in RER levels post-training (*p < 0*.05), accompanied by substantial improvements in peak oxygen consumption (peak VO_2_, *p < 0*.01) and exercise duration (*p* < 0.05). Moreover, the COPD+NAFLD group showed a significant increase in VO_2_ at the anaerobic threshold (*p* < 0.05), indicating enhanced metabolic efficiency and strengthened glycolytic capacity, which facilitates energy acquisition by muscles and ultimately improves exercise endurance. A post-training increase in RER in COPD patients can be considered a positive adaptation, reflecting improved glucose metabolism, and is typically associated with enhanced exercise tolerance. Although BMI was significantly higher in the COPD+NAFLD group at baseline and was independently associated with peak VO₂, consistent with previous reports (14, 21), this imbalance cannot be fully eliminated. However, prior studies have demonstrated that lean body mass and muscle content are more strongly associated with VO₂ than BMI, and sarcopenia is prevalent in both COPD (including the lean phenotype) and NAFLD. Therefore, it is plausible that the magnitude of training-induced improvement in VO₂ in our cohort was not substantially influenced by BMI. Our multiple regression analysis using delta VO₂ as the outcome showed no significant effect of BMI or ALT on training-induced VO₂ improvement (Supplementary Tables S1–S2).

Early Growth Response 1 (EGR1) is a zinc finger transcription factor that plays a crucial role in various physiological and pathological processes, particularly in inflammation and immune regulation. Recent studies have identified EGR1 as a key factor in the pathogenesis of COPD and NAFLD. Changes in EGR1 expression could be related to its role in cellular stress responses and immune modulation. In COPD, the upregulation of EGR1 is closely associated with increased expression of pro-inflammatory cytokines such as IL-1β and tumor necrosis factor-alpha (TNF-*α*), suggesting its role in promoting inflammation. Additionally, EGR1 is involved in regulating autophagy in COPD (22)and can bind to the MUC5AC promoter upon stimulation with cigarette smoke extract (CSE), contributing to excessive mucus secretion in COPD patients. In NAFLD, EGR1 inhibits the transcription of fatty acid oxidation genes in a peroxisome proliferator-activated receptor alpha (PPARα)-dependent manner, thereby affecting lipid metabolism. Studies have shown that liver-specific deletion of EGR1 significantly reduces glucose production, improving systemic glucose tolerance and insulin sensitivity. Moreover, EGR1 upregulation has been linked to enhanced p53 activity and increased intracellular levels of p66, which may accelerate the progression of NAFLD to liver fibrosis (4, 5, 23–26). Notably, EGR1 emerged as a hub gene in peripheral blood transcriptomic analysis and was also detected in publicly available liver single-cell RNA-seq data (GSE129516). In the NAFLD liver dataset, EGR1 expression was positively correlated with M2 macrophages and negatively correlated with monocytes, suggesting a potential link between EGR1 and macrophage polarization.

Evidence from experimental liver injury models indicates that EGR1 can exert context-dependent or even opposing functions. In chronic metabolic injury, such as NAFLD, persistent EGR1 activation promotes fibrosis through a TGF-*β*–driven program. In contrast, in acute toxic injury (e.g., CCl₄-induced fibrosis), EGR1 facilitates tissue repair by supporting normal wound-healing responses, and its absence worsens fibrosis. These findings highlight that the underlying nature of tissue injury and systemic environment shapes EGR1’s biological output. In our study, EGR1 expression was downregulated after exercise only in the COPD+NAFLD group, whereas a slight upward trend was observed in the COPD-only group. This divergence may reflect a balance between two opposing forces: acute exercise–induced stress signaling, which transiently induces EGR1, and the chronic corrective effects of training, which suppress the upstream drivers of EGR1 expression. In COPD+NAFLD, the baseline metabolic milieu—characterized by hyperinsulinemia, elevated free fatty acids, and hepatic inflammation—likely sustains high EGR1 expression via MAPK/ERK and NF-κB activation, compounded by disruption of the liver-specific HNF4α/SHP regulatory axis. Exercise training can attenuate these chronic metabolic and inflammatory stimuli, improve insulin sensitivity, and promote fatty acid oxidation (supported by the distinct change in RER at AT, ΔP = 0.039), thereby reducing transcriptional drive to EGR1. In COPD-only patients, lacking severe metabolic dysregulation, acute exercise stressors (mechanical strain, oxidative stress, transient hypoxia) may dominate the post-exercise molecular landscape, activating EGR1 through MAPK/ERK and AP-1 pathways, resulting in maintained or slightly increased expression despite functional improvement.

This study serves as a pilot investigation and has several limitations. First, the small sample size (*n* = 12, with six patients per group) significantly limits the statistical power and generalizability of our findings. In addition, this was a pilot exploratory study conducted without a pre-specified hypothesis, and no *a priori* effect size estimation or sample size calculation was performed; therefore, the statistical power to detect clinically meaningful effects is limited. Second, there is a lack of long-term follow-up data. Third, there is a lack of mechanistic studies on key hub genes. Fourth, we integrated peripheral blood transcriptomic data with publicly available liver single-cell RNA-seq data for exploratory purposes; this approach was intended solely to provide a basis for subsequent mechanistic studies in animal models or *in vitro* systems, rather than to serve as direct evidence of shared immune cell distributions between blood and liver. To address these limitations, we are recruiting more patients and have completed single-cell data analysis, with plans to proceed with further validation through animal and cell-based experiments.

## 5 Conclusion

This pilot study examined the effects of a 12-week exercise training program in COPD patients with and without NAFLD. Both groups showed measurable improvements in exercise endurance, peak oxygen consumption, and cardiopulmonary performance. The COPD+NAFLD group exhibited some differential metabolic and inflammatory changes, including reductions in selected pro-inflammatory cytokines, increases in IL-4 expression, and shifts in metabolic efficiency reflected by changes in RER at the anaerobic threshold and VO₂ at AT. Downregulation of Early Growth Response 1 (EGR1) was observed only in the COPD+NAFLD group and may be related to these adaptations. While these findings suggest potential phenotype-specific responses to exercise training, the small sample size limits definitive conclusions, and larger studies with detailed mechanistic evaluation are needed to confirm these observations.

## Data Availability

The datasets presented in this study can be found in online repositories. The names of the repository/repositories and accession number(s) can be found in the article/Supplementary material.
