# Supplementary material for: Exercise training improves metabolic and circulatory function in COPD patients with NAFLD: evidence from clinical and molecular profiling
Source: Front Med (Lausanne). 2025 Aug 29;12:1660072. doi: 10.3389/fmed.2025.1660072 (PMC12425784; doi:10.3389/fmed.2025.1660072)
Supplement: Supplementary file 1 [file Table_1.docx]

**Table S1**

| Primers | Sequence (5’-3’) |
| --- | --- |
| **RPS7** | F: ATCCAAGTCCGGCTAGTACG |
|  | R: GGCTGCCATCTAGTTTGACG |
| **NID1** | F: GACTGACCTTCGATGCGTTC |
|  | R: CAAGATCGAGAGCAACCACG |
| **FABP1** | F: AGTTTCTCCGGCAAGTACCA- |
|  | R: CCCCACCGTGAATTCGTTTT |
| **S100A4** | F: GATGAGCAACTTGGACAGCAA |
|  | R: CTGGGCTGCTTATCTGGGAAG |
| **TSC22D1** | F: AGTTTCTCCGGCAAGTACCA- |
|  | R: CCCCACCGTGAATTCGTTTT |
| **EGR1** | F: AGCGTCAGGTCCCGTTTTC |
|  | R: CTGGTGCATTGTGTTGGGT |
| **β-actin** | F: CCATCTACGAGGGCTATGCT |
|  | R: CTTTGATGTCACGCACGATT |

**Table S2.**

**Example calculation of Δ50% training workload based on CPET data**

| **Parameter** | **Value** | **Unit** | **Notes** |
| --- | --- | --- | --- |
| Anaerobic threshold (AT) | 20 | Watts (W) | Obtained from CPET |
| Peak load | 30 | Watts (W) | Maximal power output during CPET |
| Ramp rate | 10 | Watts/min | Incremental increase per minute during CPET |
| Ramp adjustment (ramp × 0.75) | 7.5 | Watts | Correction factor per protocol |
| Adjusted AT = (AT – ramp × 0.75) | 12.5 | Watts |  |
| Adjusted Peak = (Peak – ramp × 0.75) | 22.5 | Watts |  |
| Δ50% load = (Adj. AT + Adj. Peak) / 2 | 17.5 | Watts | Individualized training workload |
| **Final training load** | **17.5 ± 10** | **Watts** | Prescribed intensity for a 30-minute exercise session |
| Pedaling rate | 55 | RPM | Fixed cadence during training |
| Effective training duration | 30 | Minutes | Excluding warm-up and cool-down |
| Cool-down duration | Variable | Minutes | Continued until heart rate returned to baseline |

Note: This training protocol is primarily based on workload (Watts) rather than heart rate. Heart rate was monitored during sessions, but was not used to prescribe intensity.

**Table S3**

**Multiple Linear Regression: peak VO₂ ~ BMI + ALT**

| **Variable** | **Estimate** | **Std. Error** | **p-value** | **95% CI** |
| --- | --- | --- | --- | --- |
| Intercept | 337.8 | 431.5 | 0.45 | [-638.3, 1314] |
| BMI | **34.69** | 14.47 | **0.04** | [1.95, 67.42] |
| ALT | -11.26 | 6.88 | 0.14 | [-26.83, 4.301] |
| R2  Adjusted R2 | 0.568  0.472 |  |  |  |

**Table S4**

**Multiple Linear Regression: delta VO₂ ~ BMI + ALT**

| **Variable** | **Estimate** | **Std. Error** | **p-value** | **95% CI** |
| --- | --- | --- | --- | --- |
| Intercept | 194.0 | 71.09 | 0.02 | [33.22, 354.8] |
| BMI | -0.45 | 2.38 | 0.85 | [-5.85, 4.94] |
| ALT | -1.73 | 1.13 | 0.16 | [-4.29, 0.84] |
| R2  Adjusted R2 | 0.210  0.034 |  |  |  |

**Table S5**

**Comparison of Exercise-Induced Improvements Between COPD+NAFLD and COPD Patients**

| **Parameter** | **COPD+NAFLD**  **mean ± SD** | **COPD mean**  **± SD** | **p-value** |
| --- | --- | --- | --- |
| FEV1%pre | 0.00 ± 1.22 | 0.96 ± 3.26 | 0.229 |
| FEV1/FVC | -3.80 ± 7.29 | 6.30 ± 8.66 | 0.117 |
| RV/TLC | -0.20 ± 1.10 | 0.98 ± 15.72 | 0.460 |
| DLco | 1.60 ± 3.58 | 10.33 ± 12.88 | 0.069 |
| Exercise  time | 48.33 ± 36.84 | 38.40 ± 11.10 | 1.000 |
| Peak load | 5.00 ± 3.87 | 4.83 ± 8.30 | 0.647 |
| VO2 | 141.00 ± 42.54 | 124.83 ± 48.60 | 0.662 |
| VO2 at AT | 125.50 ± 55.97 | 74.60 ± 28.87 | 0.082 |
| RER at AT | 0.67 ± 0.52 | 0.60 ± 0.55 | 0.772 |
| O2/HR | 6.58 ± 2.46 | 5.40 ± 2.70 | 0.580 |
